# *CCNE1* Gene Amplification Might Be Associated with Lymph Node Metastasis of Gastric Cancer

**DOI:** 10.3390/genes16060617

**Published:** 2025-05-22

**Authors:** Hinano Nishikubo, Kyoka Kawabata, Tomoya Sano, Saki Kanei, Rika Aoyama, Dongheng Ma, Daiki Imanishi, Takashi Sakuma, Koji Maruo, Yurie Yamamoto, Canfeng Fan, Masakazu Yashiro

**Affiliations:** 1 Department of Molecular Oncology and Therapeutics, Osaka Metropolitan University Graduate School of Medicine, 1-4-3 Asahi-machi, Abeno-ku, Osaka 545-8585, Japan; sn23089k@st.omu.ac.jp (H.N.); sn23131y@st.omu.ac.jp (K.K.); sb24524y@st.omu.ac.jp (T.S.); sn23107b@st.omu.ac.jp (S.K.); si22406x@st.omu.ac.jp (R.A.); p24120g@omu.ac.jp (D.M.); sy23003h@st.omu.ac.jp (D.I.); so22500y@st.omu.ac.jp (T.S.); k.maruo0720@gmail.com (K.M.); yurieyamamoto917@gmail.com (Y.Y.); y23703o@omu.ac.jp (C.F.); 2 Cancer Center for Translational Research, Osaka Metropolitan University Graduate School of Medicine, 1-4-3 Asahimachi, Abeno-ku, Osaka 545-8585, Japan

**Keywords:** gastric cancer, CCNE1, lymph node metastasis, C-CAT, predictive factor, therapeutic target

## Abstract

**Background**: Lymph node (LN) metastasis is one of the most frequent metastatic patterns in patients with gastric cancer (GC); however, few genes predictive of LN status in GC have been identified. **Aims**: We aimed to identify candidate genes associated with LN metastasis by analyzing the Center for Cancer Genomics and Advanced Therapeutics (C-CAT) database and performing immunohistochemical analysis of GC cases at our hospital. **Patients and Methods**: A total of 2028 GCs from the C-CAT database were enrolled to identify genetic alterations. A total of 360 GC patients who underwent gastrectomy at our hospital were enrolled to examine the clinical significance of CCNE1 expression via an immunohistochemical study. **Results**: A total of 977 cases out of 2028 GC patients showed LN metastasis. Genetic alterations of ERBB2, CCNE1, MYC, ZNF217, and GNAS were frequent in the LN metastasis group. CCNE1-positive expression was found in 108 (30.0%) of the 360 GC samples. LN metastasis was significantly (*p* = 0.01) more frequent in CCNE1-positive patients. In addition, the CCNE1-positive group had a significantly (*p* < 0.001) poorer prognosis than the CCNE1-negative group, which was especially evident for GC patients at stage I. CCNE1 positivity was significantly (*p* < 0.001) correlated with postoperative recurrence. **Conclusions**: CCNE1 gene amplification is associated with LN metastasis of GC.

## 1. Introduction

Gastric cancer (GC) is the third most common malignancy in the world [1]. Although GC is a global disease, it has regional characteristics, and its incidence and mortality tend to be higher in Asia, including Japan. In Japan, the five-year survival rate for advanced GC after radical surgery remains poor at around 70% due to frequent metastasis of the lymph nodes (LNs), peritoneum, and liver after surgery [2]. Because LN metastasis is one of the most frequent metastatic patterns in GC patients after surgery, the prediction of LN metastasis and the development of new therapeutic targets after surgery are important to improve the prognosis of advanced GC [3,4].

Recently, multi-cancer genetic profiling testing (MGPT), which examines hundreds of cancer-associated genes (usually 100–700) to identify actionable alterations, has been widely applied for patients with advanced stages of cancers. In Japan, MGPT has started as an insurance medical treatment for refractory cancer patients from 2019. As a result, “cancer genomic medicine” has been broadly implemented for cancer patients at the advance stage [5,6]. The genomic and clinical information collected from MGPT is stored at the Center for Cancer Genomics and Advanced Therapeutics (C-CAT). MGPT facilities, academia, and companies can access this information through the data-sharing system of the C-CAT Medical-Use Portal and the C-CAT Research-Use Portal (https://www.ncc.go.jp/en/c_cat/use/index.html (accessed on 17 May 2025) [5]. The C-CAT database, which includes data for over 70,000 patients as of April 2024, is highly useful for the analysis of gene abnormalities of various types of cancers.

Cell cycle dysregulation is one of the mechanisms by which cancer cells proliferate abnormally. DNA replication, gene transcription, protein expression, and protein modification are all cell cycle-dependent, and cell cycle acceleration is also closely related to tumor formation. Cyclin E1 (*CCNE1*) is one of the oncogenes involved in the cell cycle. *CCNE1* is involved in the transition from G1 to the S phase of the cell cycle in normal and cancer cells by forming a complex with a cyclin-dependent kinase (CDK2). The cyclin E1–CDK2 complex or the cyclin D–CDK4/6 complex phosphorylates *RB1*, thereby liberating E2F [7,8,9]. *CCNE1* gene amplification has been reported in breast, ovarian, and uterine cancers and is thought to be associated with a poor prognosis. CCNE1 overexpression is also observed in GC, but its clinical significance remains unclear, and further investigation is required.

In this study, we aimed to identify gene alterations frequently occurring in GC with LN metastases using the C-CAT data, as well as analyze the relationship between CCNE1 expression and the clinicopathologic factors of GC.

## 2. Materials and Methods

### 2.1. MGPT Data from C-CAT

A total of 71,779 tumors were enrolled in the C-CAT database between June 2019 and April 2024. In this study, we analyzed 2028 GC patients from 71,779 tumors. Genetic alterations of tumors were examined using 1 of 5 MGPT methods, such as the OncoGuide™ NCC Oncopanel System (NCC) (Sysmex Co., Ltd., Kobe, Japan), FoundationOne^®^ CDx (F1CDx) (Foundation Medicine Inc., Cambridge, MA, USA), FoundationOne^®^ Liquid CDx (F1L) (Foundation Medicine Inc.), Guardant360^®^ CDx sequencing technology (G360) (Guardant Health, Palo Alto, CA, USA), and GenMineTOP^®^ Cancer Genome Profiling Systems (GMT) (KONICA MINOLTA REALM Co., Inc., Tokyo, Japan). In the 2028 GC patients, NCC was performed for 190 cases, F1CDx for 1559 cases, F1L for 238 cases, G360 for 19 cases, and GMT for 22 cases. NCC, F1CDx, and GMT analyze the cancer tissue. In contrast, F1LCDx and G360 analyze circulating tumor DNA in blood. Supplementary Figure S1 shows the public C-CAT data for GC cases.

### 2.2. Extraction of Genetic Abnormalities

MGPT detects base substitution/insertion/deletion mutations, gene amplification/deletion, and gene fusions in cancer-related genes using next-generation sequencers. Among the genes extracted by MGPT, only genetic abnormalities evaluated as pathogenic, likely pathogenic, oncogenic, or likely oncogenic in the clinical annotation of C-CAT findings were extracted. Pathogenic, likely pathogenic, oncogenic, likely oncogenic, and a variant of uncertain significance (VUS) were evaluated based on the definitions outlined in the interpretation guidelines for sequence variants established by a joint consensus recommendation from the American College of Medical Genetics and Genomics and the Association for Molecular Pathology. Oncogenic/likely oncogenic classifications follow C-CAT’s proprietary clinical significance criteria. These classifications serve as evidence demonstrating the association between genetic abnormalities and cancer, based on database analysis and research findings. VUS cases were excluded in this study.

### 2.3. GC Tissue Specimens

A total of 360 stage I, stage II, and stage III patients who had histologically confirmed primary GC at Osaka Metropolitan University Hospital were retrospectively enrolled in this study. Supplementary Figure S1 shows the hospital-based GC cases. All patients underwent a resection of the gastric tumor and regional LN at our hospital. None of the patients had received radiotherapy or chemotherapy before the surgery. The pathologic findings were defined according to the Japanese Classification of Gastric Carcinoma (15th edition) [10]. And the pathologic classifications were made according to the UICC TNM classification of malignant tumors (7th edition) [11]. This study was approved by Osaka Metropolitan University’s ethics committee (approval number 0924, 2022-077). Informed consent was obtained from all patients, and all methods were performed in accordance with relevant ethical guidelines for life science and medical research involving human subjects.

### 2.4. Immunostaining of CCNE1

Paraffin-embedded sections from 360 GC cases were incubated with 3% hydrogen peroxide to inactivate endogenous peroxidase activity. Next, these sections were heated at 105 °C for 15 min in the target solution (DAKO, Carpinteria, CA, USA), and a protein-blocking reagent (Nichirei Bioscience, Tokyo, Japan, 10% rabbit normal serum) was added to prevent nonspecific binding. The anti-CCNE1 antibody (1:100; Santa Cruz, Dallas, TX, USA) was added to the sections and incubated at 4 °C overnight. These sections reacted with the secondary antibody (Nichirei Bioscience, mouse) for 30 min at room temperature, followed by 5 min with the peroxidase-conjugated antibody. Finally, the DAB reagent was added and allowed to reacted for 5 min, and contrast staining was performed using hematoxylin. The immunostaining intensity score was rated 0–3 as follows (0 = negative, 1+ = weak, 2+ = moderate nuclear staining, and 3+ = strong nuclear staining), and positivity in cancer cells was also graded on a scale from 0 to 3 (0 = 0%, 1 = 1–69%, 2 = 70–89%, and 3 = 90–100%). The two scores were added to obtain the results with a range of 0–6. When intensity was scored 2–3, the proportion score (%) was used for the final score. When intensity was scored 2–3, the proportion score (%) was evaluated for the final score. When intensity was scored 0–1, the proportion was evaluated as 0. CCNE1 expression was finally considered positive when scores were ≥4. The evaluation was performed by two independent, double-blind observers who were unaware of the clinical data and outcomes. If a discrepancy was found between their assessments, the evaluation was rechecked and discussed.

### 2.5. Statistical Analysis

A chi-square test or Fisher’s exact test was used where appropriate. Overall survival (OS) and recurrence-free survival (RFS) were estimated using the Kaplan–Meier method, and differences between the two groups were compared using the log-rank test. Univariate and multivariate analyses were also performed using the Cox proportional hazards model. In addition, binomial logistic regression was used to analyze dichotomous dependent variables, and the Hosmer–Lemeshow goodness-of-fit test was used to examine the relationship between the two items. A *p*-value of <0.05 was considered statistically significant. All statistical analyses were performed using SPSS^®^ version 28 (IBM Corp., Armonk, NY, USA).

## 3. Results

### 3.1. Metastatic Lesions and Gene Amplification in 2028 GC Patients from the C-CAT Database

**Figure 1:**
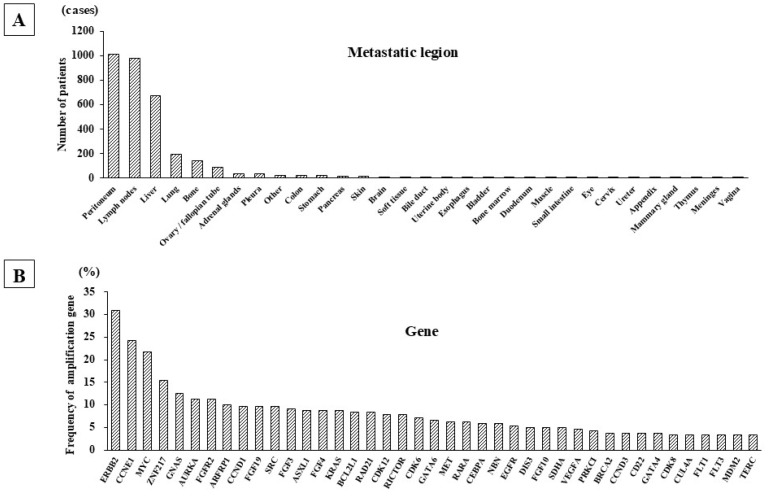
Metastatic lesions and gene amplification of GC cases from C-CAT data. (**A**) Metastatic sites of 2028 GC patients. Multi-cancer genome profiling of 2028 GC cases in the C-CAT database showed that the most frequent site of metastasis was the peritoneum, followed in order by the LN, liver, lung, and bone. (**B**) Amplified genes in GC patients with LN metastasis. The most frequently amplified gene was *ERBB2*, followed by *CCNE1* and *MYC*. *ERRB2* amplification was frequently detected in GC patients with or without LN metastasis.

Metastatic lesions were frequently found at the peritoneum, LN, liver, lungs, and ovaries/fallopian tubes in the C-CAT data of 2028 GC patients. Among the 2028 GC patients, the number of GC cases with peritoneal dissemination, LN metastasis, liver metastasis, lung metastasis, and bone metastasis were 1010 (49.8%), 977 (48.1%), 676 (33.3%), 194 (9.5%), and 141 (6.9%), respectively (Figure 1A). Among the 977 patients with LN metastasis, 240 showed metastasis only on the LN (i.e., no other recurrence sites), and these patients showed frequent genetic alterations in *ERBB2*, *CCNE1*, *MYC*, *ZNF217*, *GNAS*, *AURKA*, *FGFR2*, and *ARFRP1* (Figure 1B). Mutations in *ERBB2*, *CCNE1*, and *MYC* were particularly frequent, being found in over 20% of these patients with exclusive LN involvement. Among the total group of 977 patients with LN metastasis with/without involvement at other sites, mutations of *TP53*, *ARID1A*, and *APC* and gene deletions in *CDKN2A*, *CDKN2B*, and *PTEN* were frequently found. In contrast, rearrangements in *EGFR*, *NOTCH1*, and *RARA* were rare (26 out of 977 patients: 2.6%).

### 3.2. Correlation Between CCNE1 Expression and Clinicopathologic Features in 360 Patients with GC

**Figure 2:**
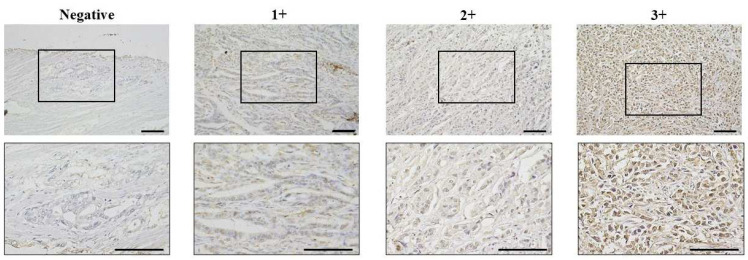
Representative pictures of CCNE1 expression in GC. CCNE1 expression was evaluated via nuclear staining of cancer cells. Lower images enlarged pictures of black box in upper images. Brightness was evaluated on a scale from 0 to 3 (0 = negative, 1+ = weak, 2+ = moderate at the nuclear, and 3+ = strong at the nuclear). CCNE1 expression was considered positive when scores were ≥4. Bar = 100 μm.

We next performed immunohistochemical analysis on tissue samples from 360 GC patients treated at our hospital. The results showed that the main site of CCNE1 expression was the nucleus of GC cells (Figure 2). Among the 360 GC cases, CCNE1 expression was found in 108 cases. The correlation between CCNE1 expression and clinicopathologic features is shown in Table 1. CCNE1 positivity was significantly correlated with gender (*p* = 0.04), the T stage (*p* = 0.01), LN metastasis (*p* = 0.01), lymphatic invasion (*p* < 0.001), recurrence (*p* < 0.001), and the clinical stage (*p* = 0.04) (Table 1).

### 3.3. Correlation Between CCNE1 Expression and Patient Survival

**Figure 3:**
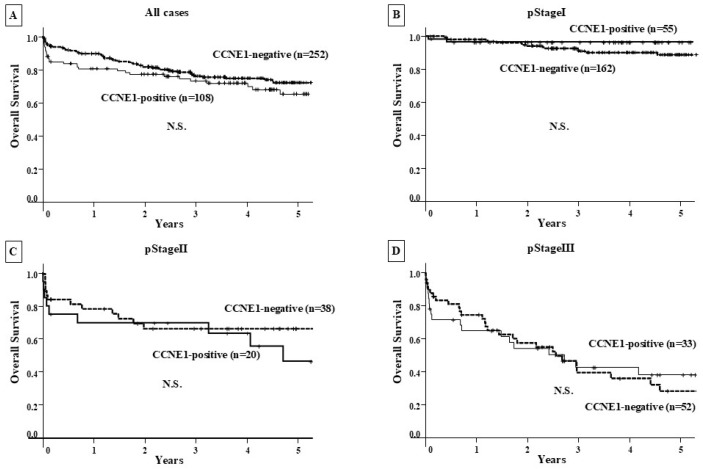
Overall survival curve. (**A**–**D**) Prognostic comparison between the CCNE1-positive and CCNE1-negative groups at each clinical stage showed no significant difference.

**Figure 4:**
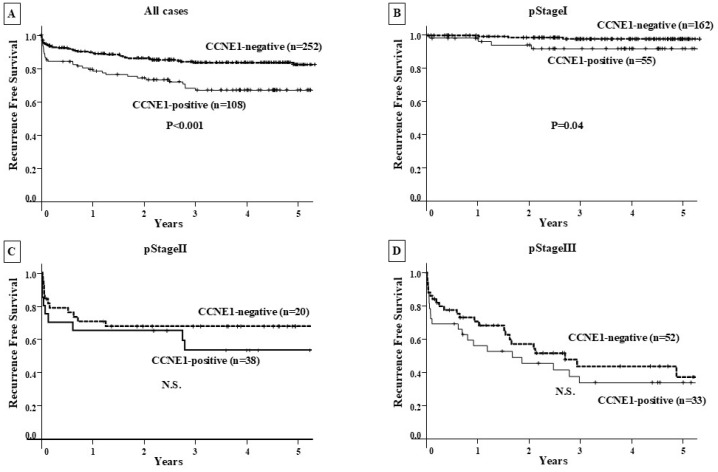
Recurrence-free survival curve. (**A**) RFS was compared between the CCNE1-positive and CCNE1-negative groups in all patients. The CCNE1-positive group had a significantly (*p* < 0.001) worse prognosis than the CCNE1-negative group. (**B**) The prognosis of CCNE1-positive and CCNE1-negative patients was compared by clinical stage. CCNE1-positive patients had a significantly poorer RFS than CCNE1-negative patients (*p* = 0.04). (**C**,**D**) The prognostic comparison between the CCNE1-positive and CCNE1-negative groups at clinical stage II/Ⅲ showed no significant difference.

There was no significant difference in OS among CCNE1-positive GC patients (n = 108) compared with CCNE1-negative patients (n = 252) at all clinical stages and at each clinical stage (Figure 3). In contrast, CCNE1-positive GC patients showed a significantly poorer RFS than CCNE1-negative patients (*p* < 0.001), and significance was observed for stage I CCNE1-positive GC patients (*p* = 0.04) (Figure 4).

The univariate analysis revealed that the RFS was significantly correlated with CCNE1 expression, inf, the T stage, LN metastasis, distant metastasis, lymphatic invasion, and venous invasion. Among these clinicopathologic factors, CCNE1 expression (*p* < 0.05), inf (*p* = 0.01), the T stage (*p* < 0.001), LN metastasis (*p* < 0001), and venous invasion were significantly correlated with RFS according to multivariate analysis (Table 2).

As a result of binary logistic regression analysis, significant differences were observed between patients with postoperative LN recurrence, CCNE1 expression (*p* = 0.03), and venous invasion (*p* < 0.001) (Table 3).

## 4. Discussion

In this study, we analyzed both the genetic alteration of 2028 GC cases from the C-CAT database of MGPT and CCNE1 expression using 360 GC tissues from a hospital. The analysis of 2028 GC cases using the C-CAT database revealed that GC frequently metastasizes to the peritoneum, LN, or liver, as previously reported [12]. These GC cases showed frequent gene amplification of *ERBB2*, *CCNE1*, and *MYC*. *CCNE1* amplification was the second most frequent amplification in GC at the advanced stage. Since the correlation between *HER2* or *MYC* amplification and LN metastasis of GC has previously been reported, the analysis of ERBB2 and MYC was excluded in this study. In contrast, no report was found about the correlation between CCNE1 and LN metastasis. Then, we focused on CCNE1. While *CCNE1* amplification has been reported to be associated with liver metastasis [13], MGPT data from C-CAT suggested that *CCNE1* amplification might play an important role for not only liver metastasis but also LN metastasis in GC.

Next, we immunohistochemically examined the clinical significance of CCNE1 expression using the GC tissue samples of 360 patients from our hospital. High CCNE1 expression was found in 108 (30.0%) of the 360 GC samples. CCNE1 expression was significantly correlated with gender, the T factor, LN metastasis, lymphatic invasion, recurrence, and the clinical stage among clinicopathologic factors. LN metastasis was significantly more frequent in CCNE1-positive cases than in CCNE1-negative case. Lymphatic invasion also showed a significant positive association with CCNE1 expression. These findings also suggest that *CCNE1* amplification might play an important role for the LN metastasis process of GC.

The OS analysis using Kaplan–Meier survival curves showed no correlation between high CCNE1 expression and a poor prognosis in the CCNE1-positive group compared with the CCNE1-negative group at both the preclinical stage and at each clinical stage. On the other hand, the CCNE1-positive group had a poorer prognosis than the CCNE1-negative group in terms of RFS for all patients. Especially at clinical stage I, the CCNE1-positive group showed a significant correlation with postoperative recurrence. *CCNE1* amplification may be closely associated with lymphatic metastasis when compared to hematogenous metastasis. Univariate and multivariate analyses using Cox regression analysis revealed that CCNE1 expression was an independent poor prognostic factor for RFS. Furthermore, binomial logistic regression analysis also revealed that CCNE1 expression was an independent poor prognostic factor for postoperative LN recurrence. These results suggest that CCNE1 expression may function as a risk factor for postoperative LN recurrence.

While one report about the correlation between CCNE1 amplification and the lymphatic invasion of GC is available, the molecular mechanism and its correlation have not yet been clarified. In contrast, in breast cancer, LN metastasis is often observed at the early stage of patients with CCNE1 amplification and Rb1 deletion [14,15]. This suggests that abnormalities in cell cycle signals, including CCNE1, may be involved in the early stage of metastasis. It has been reported that CCNE1 regulates cyclin E1 in the cell cycle from G1 to the S phase. *CCNE1* amplification may result in the promotion of the transcriptional induction of S-phase-related genes such as cyclin A and accelerate the G1 phase, causing cell cycle dysregulation and contributing to cancerization [16], suggesting that CCNE1 accumulation in the early stage of GC may promote the LN metastasis process via the induction of the G1 phase to the S phase. To our knowledge, this study is the first to suggest that CCNE1 is an important molecule for LN metastasis of carcinomas.

The above findings suggested that the development of a CCNE1 inhibitor might be useful for molecular target therapy against GC with LN metastasis. Currently, CDK4/6 inhibitors, CDK2 inhibitors, wee1 inhibitors, etc., are available as treatments for CCNE1. There are small-molecule CDK4/6 inhibitors such as palbociclib, abemaciclib, and ribociclib that have already been approved for use in breast cancer, and clinical trials are currently underway in Japan and overseas for other cancers [17]. In a phase 3 clinical trial of palbociclib (CDK4/6 inhibitor) for advanced breast cancer, it was revealed that CCNE1 amplification was observed in CDK4/6 inhibitor-resistant cells, and it has been reported that when CDK2 inhibitors were administered to these cells, sensitivity to CDK4/6 inhibitors was maintained [18,19,20,21]. Therefore, if CDK4/6 inhibitors become available as treatments for CCNE1, it may be possible to use CDK2 inhibitors to treat CCNE1 even when resistance develops.

No clinical trial targeting CCNE1 is found to the best of our knowledge. CCNE1 might be a promising target for GC patients with a high risk of LN metastasis. GC with CCNE1 amplification is common in Japan, and a correlation with LN metastasis has been observed, suggesting that CCNE1 inhibitors may be a promising target for GC treatment.

In conclusion, CCNE1 amplification correlates with GC and LN metastasis and may be a predictor of postoperative LN recurrence.

## Figures and Tables

**Table 1:** Correlation between CCNE1 expression and clinicopathologic factors in gastric cancer patients.

Clinicopathologic Features		CCNE1 Staining		
		Negative	Positive	*p* Value
		N = 252	N = 108	
Age	<65	102 (69.9%)	44 (30.1%)	n.s.
	≥65	150 (70.1%)	64 (29.9%)	
Gender	female	78 (78.0%)	22 (22.0%)	0.04
	male	174 (66.9%)	86 (33.1%)	
Macroscopic type	Borrmann type 4	11 (78.6%)	3 (21.4%)	n.s.
	other types	241 (69.7%)	105 (30.3%)	
Microscopic type	differentiated	136 (69.4%)	60 (30.6%)	n.s.
	undifferentiated	116 (70.7%)	48 (29.3%)	
Inf	α and β	200 (70.7%)	83 (29.3%)	n.s.
	γ	52 (67.5%)	25 (32.5%)	
T stage	T1	131 (76.2%)	41 (23.8%)	0.01
	T2,3,4	121 (64.4%)	67 (35.6%)	
Lymph node metastasis	negative	161 (75.6%)	52 (24.4%)	0.01
	positive	91 (61.9%)	56 (38.1%)	
Lymphatic invasion	negative	128 (80.0%)	32 (20.0%)	<0.001
	positive	124 (62.0%)	76 (38.0%)	
Venous invasion	negative	214 (70.4%)	90 (29.6%)	n.s.
	positive	38 (67.9%)	18 (32.1%)	
Recurrence	negative	213 (74.0%)	75 (26.0%)	<0.001
	positive	39 (54.2%)	33 (45.8%)	
Clinical stage	Ⅰ & Ⅱ	200 (72.7%)	75 (27.3%)	0.04
	Ⅲ	52 (61.2%)	33 (38.8%)	

CCNE1 was positive in 108 cases out of the 360 GC cases (30.0%) in this study. LN metastasis was found in 56 cases (51.9%) in the CCNE1-positive group, but the CCNE1-negative group did not showed LN metastasis in most cases (n = 161, 63.9%). Six factors including gender (*p* = 0.04), the T stage (*p* = 0.01), LN metastasis (*p* = 0.01), lymphatic invasion (*p* < 0.001), recurrence (*p* < 0.001), and the clinical stage (*p* = 0.04) were significantly correlated with amplified CCNE1. They were indicated to be associated with amplified CCNE1, LN metastasis, and lymphatic invasion according to the statistical analysis.

**Table 2:** Univariate and multivariate analyses with respect to recurrence-free survival.

Parameter	Univariate Analysis			Multivariate Analysis		
	Hazard Ratio	95% CI	*p*-Value	Hazard Ratio	95% CI	*p*-Value
CCNE1						
positive vs. negative	2.12	1.34–3.38	<0.001	1.63	1.01–2.63	<0.05
Age						
<65 vs. ≥65	1.19	0.74–1.92	n.s.			
Gender						
female vs. male	0.90	0.54–1.49	n.s.			
Macroscopic type						
Borrmann’s type 4 vs. Other types	5.84	2.88–11.84	<0.001	1.72	0.81–3.69	n.s.
Microscopic type						
differentiated vs. undifferentiated	2.02	1.26–3.25	<0.001	1.24	0.73–2.13	n.s.
Inf						
α and β vs. γ	3.18	1.99–5.09	<0.001	2.09	1.24–3.51	0.01
T stage						
T1 vs. T2–T4	15.46	6.23–38.38	<0.001	4.72	1.76–12.61	<0.001
LN metastasis						
negative vs. positive	6.84	3.87–12.09	<0.001	2.71	1.47–4.99	<0.001

**Table 3:** Analysis of factors associated with postoperative lymph node recurrence using binary logistic regression.

Parameter	Unifactorial Analysis			Final Regression Model		
	Hazard Ratio	95% CI	*p*-Value	Hazard Ratio	95% CI	*p*-Value
CCNE1						
positive vs. negative	2.74	1.13–6.67	0.03	2.89	1.10–7.55	0.03
Age						
<65 vs. ≥65	0.49	0.20–1.20	0.12	0.43	0.16–1.11	n.s.
Gender						
female vs. male	0.68	0.44–3.50	n.s.			
Microscopic type						
differentiated vs. undifferentiated	0.89	0.37–2.17	n.s.			
Inf						
α and β vs. γ	1.16	0.41–3.27	n.s.			
T stage						
T1 vs. T2,3,4	4.18	1.38–12.67	0.01	1.96	0.57–6.71	n.s.
Venous invasion						
negative vs. positive	7.19	2.89–17.89	<0.001	5.64	2.01–15.79	<0.001

## Data Availability

The data that support the findings will be available in https://www.ncc.go.jp/jp/c_cat/use/index.html (accessed on 30 April 2024).

## Supplementary Materials

The following supporting information can be downloaded at: https://www.mdpi.com/article/10.3390/genes16060617/s1, Figure S1: The public data and hospital-based cases. A total of 71,779 cancer cases were enrolled from C-CAT public data. Gastric cancer was 2028 cases in all 71,779 cases. Among 2028 GC cases, 977 cases were LN metastasis positive and 1051 cases were negative. In GC with LN metastasis, CCNE1 gene amplification was observed in 234 cases and no amplification was observed in 743 cases. Immunohistochemical study was performed using GC tissue samples from 360 patients treated at Osaka Metropolitan University Hospital. Among the 360 GC patients 108 cases were CCNE1 positive and 252 cases were negative.

## Abbreviations

lymph node (LN), gastric cancer (GC), Center for Cancer Genomics and Advanced Therapeutics (C-CAT), comprehensive genomic profiling (CGP), cyclin-dependent kinase (CDK2), OncoGuide™ NCC Oncopanel System (NCC), FoundationOne^®^ CDx (F1CDx), FoundationOne^®^ Liquid CDx (F1L), Guardant360^®^ CDx sequencing technology (G360), GenMineTOP^®^ Cancer Genome Profiling Systems (GMT), overall survival (OS), and recurrence-free survival (RFS).
