# Supplementary material for: CCNE1 Gene Amplification Might Be Associated with Lymph Node Metastasis of Gastric Cancer
Source: Genes (Basel). 2025 May 22;16(6):617. doi: 10.3390/genes16060617 (PMC12191482; doi:10.3390/genes16060617)
Supplement: Supplementary file 1 [file genes-16-00617-s001.zip › genes-3634918-supplementary.pptx]

## Slide 1
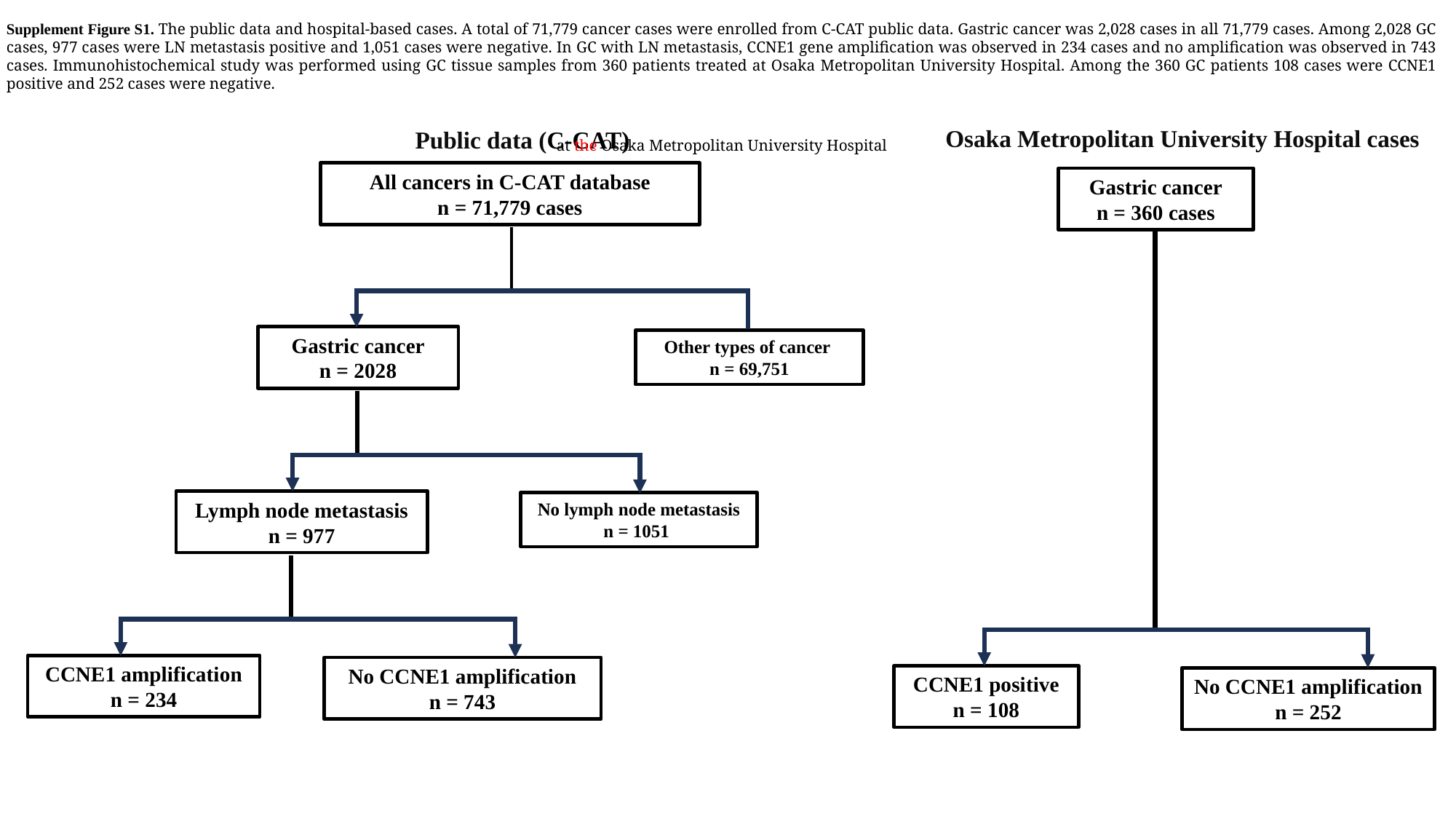

Supplement Figure S1. The public data and hospital-based cases. A total of 71,779 cancer cases were enrolled from C-CAT public data. Gastric cancer was 2,028 cases in all 71,779 cases. Among 2,028 GC cases, 977 cases were LN metastasis positive and 1,051 cases were negative. In GC with LN metastasis, CCNE1 gene amplification was observed in 234 cases and no amplification was observed in 743 cases. Immunohistochemical study was performed using GC tissue samples from 360 patients treated at Osaka Metropolitan University Hospital. Among the 360 GC patients 108 cases were CCNE1 positive and 252 cases were negative.
Osaka Metropolitan University Hospital cases
Public data (C-CAT)
at the Osaka Metropolitan University Hospital
All cancers in C-CAT database
n = 71,779 cases
Gastric cancer
n = 360 cases
Gastric cancer
n = 2028
Other types of cancer
n = 69,751
Lymph node metastasis
n = 977
No lymph node metastasis
n = 1051
CCNE1 amplification
n = 234
No CCNE1 amplification
n = 743
CCNE1 positive
n = 108
No CCNE1 amplification
n = 252
